# The kynurenine and serotonin pathway, neopterin and biopterin in depressed children and adolescents: an impact of omega-3 fatty acids, and association with markers related to depressive disorder. A randomized, blinded, prospective study

**DOI:** 10.3389/fpsyt.2024.1347178

**Published:** 2024-02-13

**Authors:** Lucia Ilavská, Marcela Morvová, Zuzana Paduchová, Jana Muchová, Iveta Garaiova, Zdenka Ďuračková, Libuša Šikurová, Jana Trebatická

**Affiliations:** ^1^ Department of Nuclear Physics and Biophysics, Faculty of Mathematics, Physics and Informatics, Comenius University, Bratislava, Slovakia; ^2^ Institute of Medical Chemistry, Biochemistry and Clinical Biochemistry, Faculty of Medicine, Comenius University, Bratislava, Slovakia; ^3^ Research and Development Department, Cultech Ltd., Port Talbot, United Kingdom; ^4^ Department of Paediatric Psychiatry, Faculty of Medicine of Comenius University and The National Institute of Children’s Diseases, Bratislava, Slovakia

**Keywords:** depressive disorder, kynurenine and serotonin pathways, neopterin, biopterin, oxidative stress, omega-3 fatty acids, children and adolescents

## Abstract

Depressive disorder is a severe mental condition. In addition to genetic factors, immunological-inflammatory factors, oxidative stress, and disturbances in neurotransmitter metabolism, kynurenine and serotonin pathways may play a role. The exact mechanisms, especially in depressed children and adolescents, are not fully understood. Our primary hypothesis was whether the metabolites of tryptophan degradation in children and adolescents with depressive disorder might be influenced by omega-3 FAs compared to omega-6 FAs during a 12-week supplementation. A secondary hypothesis was to investigate whether tryptophan metabolites in children and adolescents are associated with markers of inflammatory response, oxidative stress, cortisol, and the serum omega-6/omega-3 FA ratio. Metabolites of tryptophan degradation and pteridines, neopterin, and biopterin in urine were analyzed with an HPLC system. Surprisingly, omega-3 FAs stimulated both kynurenine (kynurenine/tryptophan ratio) and serotonin (5-hydroxytryptophan) pathways, whereas omega-6 FAs only increased the kynurenine/tryptophan ratio. Neopterin and biopterin were not different from the healthy controls. Biopterin increased after omega-3 FA supplementation. Serotonin was positively correlated with lipoperoxidation and a marker of oxidative protein damage. Of the monitored tryptophan metabolites, only 5-hydroxyindolacetic acid was positively correlated with the severity of depression, total cholesterol, and negatively with brain-derived neurotrophic factor and glutathione peroxidase. In conclusion, in children and adolescents, both supplemented FAs stimulated the kynurenine pathway (kynurenine/tryptophan ratio) and kynurenine formation. However, the serotonin pathway (5-hydroxytryptophan) was stimulated only by omega-3 FA. Tryptophan metabolism is associated with oxidative stress, inflammation, total cholesterol, and cortisol. We are the first to point out the association between the kynurenine pathway (KYN/TRP ratio) and the omega-6/omega-3 FA ratio. The metabolite 5-HIAA could play a role in the pathophysiology of depressive disorder in children and adolescents.

**Clinical Trial Registration:**

https://www.isrctn.com/ISRCTN81655012, identifier ISRCTN81655012.

## 1 Introduction

The depressive disorder (DD) in children and adolescents is a significant public health concern, given its detrimental outcomes that can result in serious health and socio-economic consequences in adulthood. The prevalence of depressive disorder in adolescents is approximately 5.7%, with a female-to-male ratio of 1.3:1 (1, 2). The primary symptoms of childhood depression include persistent sadness, loss of interest and joy, increased fatigue, sleep problems, or suicidal attempts (3). The precise causes of the onset and development of a depressive disorder are not fully understood. In addition to genetic factors, disorders in neurotransmitter metabolism, an inflammatory response to various stimuli, activation of the hypothalamic-pituitary-adrenal axis (HPA), oxidative stress, insufficient levels of omega-3 fatty acids (FA), disturbances in cell membrane composition (fluidity), and other factors can contribute to the development of a depressive disorder. Specifically, an imbalance in the catabolism of the amino acid tryptophan is a focal point of research interest (4–7).

**Figure 1:**
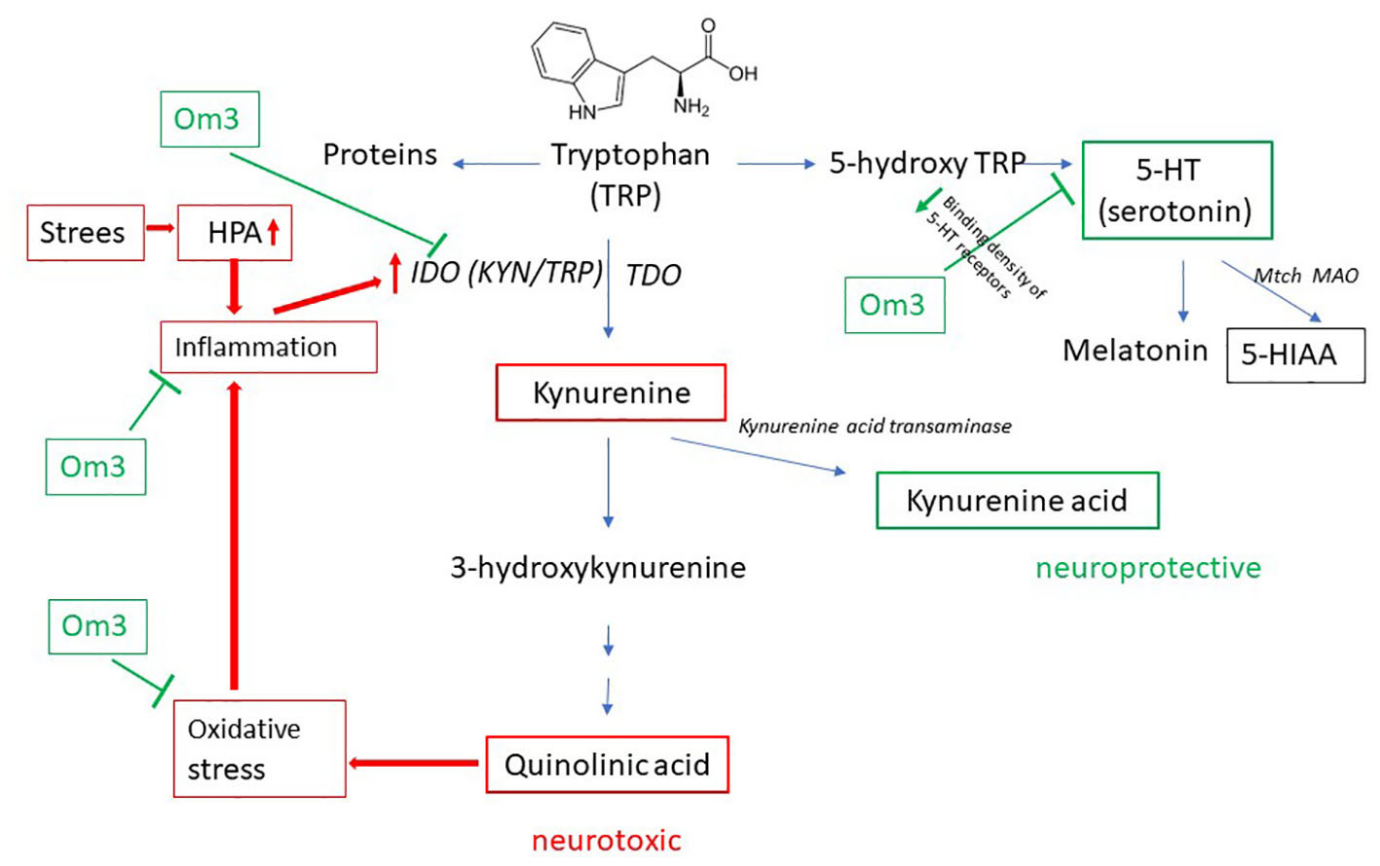
Catabolism of TRP. TDO, tryptophan 2,3-dioxygenase; IDO, indoleamine 2,3-dioxygenase; 5-HT, 5-hydroxytryptamine; 5-HIAA, 5-hydroxyindolacetic acid; Om3, omega-3 fatty acid; HPA, hypothalamic-pituitary-adrenal axis.

Tryptophan (TRP) is an essential amino acid obtained from food. It can be catabolically degraded via the kynurenine or serotonin pathway (**Figure 1**). TRP undergoes oxidation by the rate-limiting enzymes indoleamine 2,3-dioxygenase (IDO, found in various organs, including nerve cells) or tryptophan 2,3-dioxygenase (TDO, present in the liver), leading to the formation of kynurenine (KYN), 3-hydroxykynurenine (3-HK), and other products, ultimately resulting in quinolinic acid (QUIN) (8, 9).

Serotonin (SER, also known as 5-hydroxytryptamine, 5-HT) is a neurotransmitter synthesized in small amounts from TRP in the brain and approximately 90% in the gastrointestinal tract (10). TRP is hydroxylated to 5-hydroxytryptophan (5-HTP) and further decarboxylated to SER. In the brain, the unstable SER is rapidly metabolized by mitochondrial monoamine oxidase (MAO) to the final and stable product – 5-hydroxyindole acetic acid (5-HIAA) (**Figure 1**). The formation of SER from TRP is crucial for the physiological transmission of signals between neurons. One hypothesis regarding depressive disorders is based on a decrease in serotonergic activity (11).

A decrease in the level of SER can be caused by a decrease in consumption of food rich in proteins, or by redirecting the catabolism of tryptophan to the kynurenine pathway. As MAO activity increases in depressed patients, the level of 5-HIAA increases and the level of SER decreases (12).

Oxidative stress is closely associated with inflammation (13), activating IDO and consequently triggering KYN production. Stress is another initiator of the kynurenine pathway: it activates the HPA axis, which in turn initiates the production of pro-inflammatory mediators such as cortisol (14, 15) and thereby activates the IDO enzyme (**Figure 1**).

KYN can also produce kynurenic acid (KA), which under physiological conditions, is attributed to neuroprotective effects. KA is a glutamate receptor antagonist that blocks the NMDA receptor of glutamate and acts as a scavenger of free radicals, such as the hydroxyl radical or superoxide (16). Under physiological conditions, there must be a balance between the formation of QUIN and KA, as QUIN is neurotoxic and KA neuroprotective in low micromolar concentrations (8, 17, 18).

In the pathophysiology of depressive disorders, the involvement of the kynurenine pathway is more likely when reactive oxygen species are generated from 3-HK and QUIN acid. Increased oxidative stress activates inflammatory pathways, leading to the production of pro-inflammatory mediators and the activation of IDO. This amplifies the pathophysiology of tryptophan degradation through the kynurenine pathway (19). The activity of IDO is also initiated by psychological stress through the HPA axis and inflammation (**Figure 1**).

The relationship between kynurenine or serotonin pathway metabolites and depressive disorder in children and adolescents is not fully understood.

Neopterin (NEO), a marker of cell-mediated immunity, and biopterin (BIO), an oxidation product of tetrahydrobiopterin (BH4), belong to the group of pteridines (20). Inflammation, specifically through the cytokine interferon-gamma, stimulates not only IDO activity and TRP catabolism but also the production of neopterin. Neopterin is non-enzymatically synthesized after inflammatory activation in monocytes, macrophages, and dendritic cells. In contrast, BH4 can be formed through the reduction of BIO. It also serves as a cofactor for nitric oxide synthases, which produce NO.

The pathways of tryptophan (TRP) catabolism and pteridine metabolism have been extensively studied in relation to regression, primarily in depressed adults. However, in children and adolescents, knowledge about these processes is lacking.

In our previous study (7), we observed that omega-3 fatty acids, in contrast to omega-6 fatty acids, reduced the severity of depression in children and adolescents. Our primary hypothesis was whether the metabolites of tryptophan degradation in children and adolescents with depressive disorder could be influenced by omega-3 fatty acids compared to omega-6 fatty acids during a 12-week supplementation. A secondary hypothesis was to investigate whether tryptophan metabolites in the kynurenine pathway (KYN and the KYN/TRP ratio representing IDO activity), the serotonin pathway (5-HTP, SER, and 5-HIAA), as well as pteridines (neopterin and biopterin) in depressed children and adolescents are associated with markers of inflammatory response, oxidative stress, cortisol, and the serum omega-6/omega-3 fatty acid ratio.

## 2 Materials and methods

This work is a part of the DEPOXIN project (ISRCTN81655012). A detailed description of the study design, recruitment of patients, inclusion and exclusion criteria, interventions, and previous study results are presented in recent publications (7, 15, 21–23). For readers’ convenience, key information about the design of the project is given in Supplements as Supplement - Study design.

### 2.1 Subjects

Briefly, 60 patients with depressive disorder aged 10-18 years and 20 age- and gender-matched healthy controls were included in this randomized, blinded, prospective study. 31 patients with depressive disorder (DD) and 29 patients with mixed anxiety and depressive disorder (MADD) were registered at the Department of Paediatric Psychiatry of the Faculty of Medicine of Comenius University and The National Institute of Children’s Diseases in Bratislava and met the inclusion criteria (age 8-18 years, diagnosis of DD or MADD). Diagnosis were established according to the ICD-10 (International Classification of Diseases).

Inclusion criteria included the diagnosis of depressive disorder (F32.0, F32.1, F32.2) or mixed anxiety and depressive disorder (F41.2), age 8-18 years, with normal eating habits and no indication of chronic somatic disease. The diagnoses were determined according to the International Classification of Diseases, 10^th^ edition (ICD 10).

Exclusion criteria were chronic somatic diseases (endocrine, metabolic, autoimmune), dietary restrictions (vegetarians, lactose intolerance, celiac disease), psychotic disorders, eating disorders, substance use disorders, personality disorders, organic mental disorders and pervasive developmental disorders.

**Figure 2:**
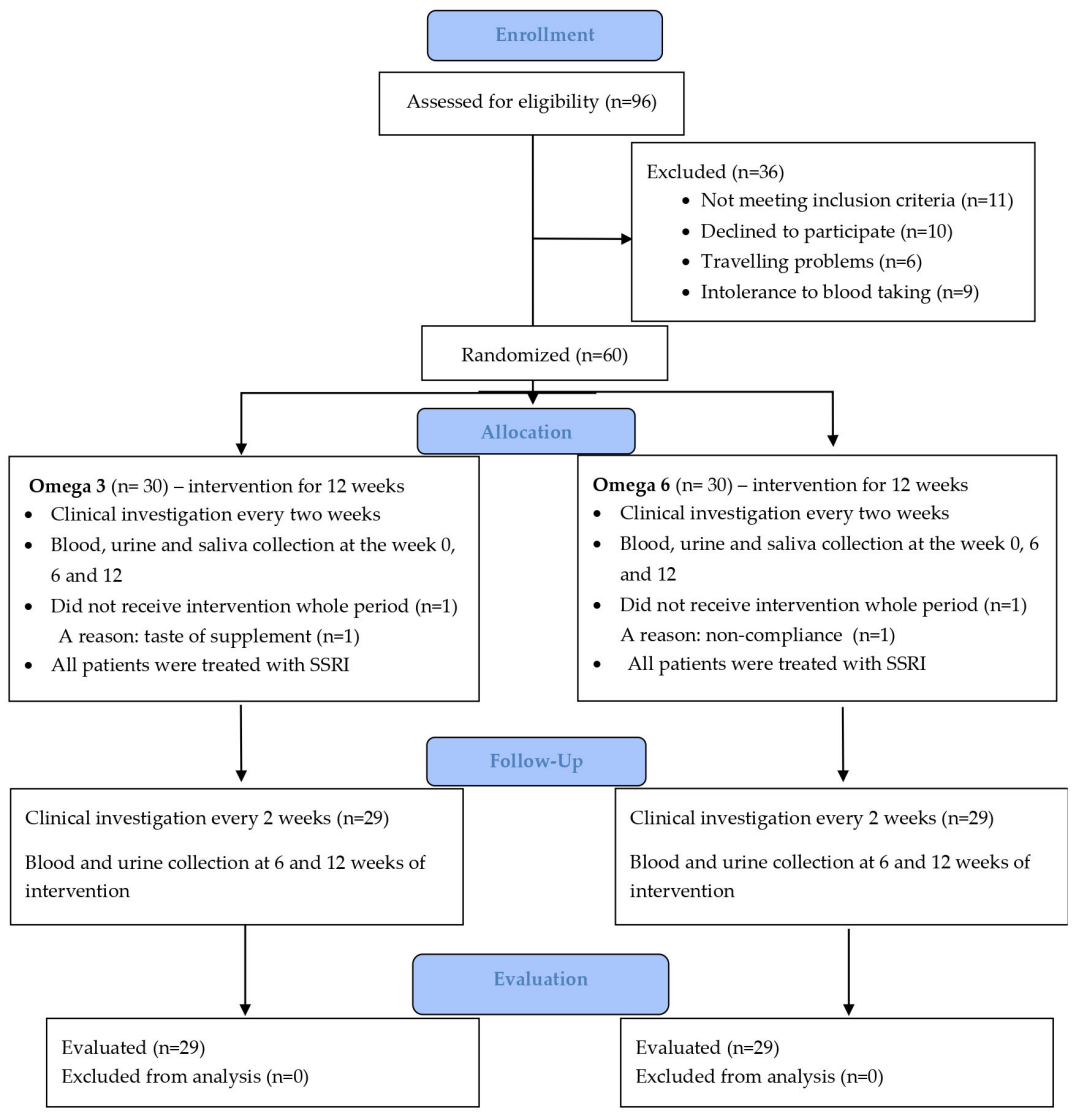
CONSORT flow diagram.

Healthy children registered at the Pediatric Center Juvenalia, Dunajská Streda, Slovakia were included in the control group. The control group did not use any supplements. Blood, urine and saliva were collected from healthy children and adolescents at the time of their inclusion in the study (Week 0). All healthy girls in the control group were menstruating. From the patient group of 48 girls, 47 were menstruating. The process of enrolment of patients is shown in the CONSORT flow diagram (**Figure 2**).

#### 2.1.1 Institutional review board statement

The human study was performed in accordance with the ethical standards outlined in the 1975 Declaration of Helsinki and its later amendments (2013). The study was approved by the Ethics Committee of the National Institute of Children’s Diseases and the Faculty of Medicine, Comenius University Bratislava (20 March 2013).

#### 2.1.2 Informed consent statement

Written informed consent was obtained from parents or legal guardians and children provided verbal assent before participation in the study. Details that might disclose the identity of the subject under study were omitted.

### 2.2 Study intervention

Patients were randomly divided into two groups. One group, referred to as the Om3 group, received omega-3 FA, while the other group, referred to as the Om6 group, received omega-6 FA as an active comparator/placebo. Both groups received these supplements in addition to their standard treatment with antidepressants, specifically selective serotonin reuptake inhibitors (SSRI). The omega-3 FA was sourced from a fish oil emulsion (Cultech Ltd, Port Talbot, UK), with a daily dose containing 2400 mg of total omega-3 FA (1000 mg of eicosapentaenoic acid (EPA) and 750 mg of docosahexaenoic acid (DHA)), maintaining an EPA : DHA ratio of 1.33:1. The omega-6 FA was sourced from a sunflower oil emulsion (Cultech Ltd, Port Talbot, UK), with a daily dose comprising 2467 mg of linoleic acid in triacylglycerol form. The dose of omega-3 FA used was determined based on a review of the literature. Patients in both groups (Om3 and Om6) received a daily dose of 20 mL of emulsion during the 12 weeks of supplementation.

### 2.3 Biological material sampling

Blood, urine, and saliva were collected from patients at the baseline (week 0) and after 6 weeks (week 6) and 12 weeks of supplementation (week 12).

Venous blood was collected from patients and healthy controls after a 12-h overnight fast. Serum and plasma were obtained by centrifuging the blood (1200 g, 10 min) with or without EDTA as an anticoagulant within 1 h of blood collection. To the remaining volume of whole blood with anticoagulant, primarily containing erythrocytes, a saline solution (0.15 mol/L NaCl) was added. This was followed by centrifugation at 1200g for 7 minutes, and the procedure was repeated two more times. The serum and plasma were aliquoted and stored at −80°C until further use. Washed erythrocytes were diluted 1:3 with chilled distilled water. After 15 min, the hemolysate was stored at −20°C until further use. In the hemolysate, hemoglobin was determined by the Drabkin method and expressed in g/L.

Second-morning urine samples were collected by spontaneous micturition and stored at −28°C. For analysis, each urine sample was thawed at room temperatures and centrifuged at 3000 g for 10 min, followed by filtration of supernatant through a 0.22 μm membrane Syringe Filter PTFE (AZ Chrom s.r.o., Bratislava, Slovak Republic).

Saliva samples were collected from each subject to determine cortisol concentrations. Sampling took place in the morning and at midday. Regarding the group of healthy children, saliva samples were taken in the morning and at midday at the baseline without any intervention. Children were asked to gently chew cotton swabs from Salivette sampling tubes (Salivette^®^ device, Sarstedt, UK) for 1 min. The samples were stored at −20°C until analyzed. Details of saliva collection are described in our paper (15).

### 2.4 Markers of the kynurenine and serotonin pathways, neopterin, biopterin, and creatinine in urine

From an ethical standpoint, we opted for non-invasively collected urine (in contrast to invasive blood collection) to determine TRP degradation metabolites, considering the observed concordance between peripheral levels and levels in the central nervous system (24).

Levels of urine metabolites (creatinine, TRP, KYN, 5-HTP, SER, 5-HIAA, NEO, BIO) were determined using reversed-phase high-performance liquid chromatography (RP-HPLC system, Prominence 20A, Shimadzu Corporation, Japan) equipped with UV-VIS absorption (module SPD-20A) and fluorescence (module RF-10AXL) detectors. The system temperature was adjusted to 25°C. LC solution software (Shimadzu Co., Kyoto, Japan) was used for analysis and processing of chromatograms.

To determine the concentration of tryptophan and its metabolites, a Nucleosil 100-5 C18 column (150 × 4.6 mm; 5 µm particle size) (Macherey-Nagel, Düren, Germany), mobile phase KH_2_PO_4_ with a concentration of 10 mmol/L and 4% methanol, pH 7.0 and a flow rate of 1 mL/min. TRP, 5-HTP, SER, 5-HIAA were detected using a fluorescence detector with an excitation wavelength of 280 nm and emission at 340 nm. Creatinine and KYN were detected using a UV-VIS absorption detector at 240 nm. To determine the concentration of neopterin and biopterin, a alphaBond™ C18 column (150 × 3.9 mm; 10 µm particle size) (Supelco, USA), mobile phase KH_2_PO_4_ with a concentration of 10 mmol/L and 3% methanol, pH 2.5 and a flow rate of 0.8 mL/min. were applicated. NEO and BIO were detected using a fluorescence detector with excitation wavelength of 350 nm and emission at 450 nm.The concentrations of the studied metabolites were determined based on the areas of the peaks using external calibration curves. The concentrations of metabolites were expressed in μmol/mmol creatinine.

### 2.5 Clinical assessment of patients’ condition

The severity of depressive disorder symptoms expressed as CDI scores (the Children’s Depression Inventory) was assessed every two weeks before and during the intervention by the study psychiatrist. A higher CDI score corresponds to a worse clinical condition for the patient. The results of the evaluation of CDI scores, the omega-6/omega-3 FA ratio, as well as the effects of fatty acids on symptoms in the same cohort of patients have already been published elsewhere (7).

### 2.6 Markers of oxidative stress, inflammation, and other indicators

Oxidative stress and inflammatory markers, BDNF, fatty acids, and cortisol were determined in the same cohort of patients as follows: (i) 8-isoprostanes (8-IsoP), the inflammation marker thromboxane (TXB) in urine; (ii) lipoperoxides (LP), advanced oxidative protein products (AOPP), nitrotyrosine (NT), trolox equivalent antioxidant capacity (TEAC), homocysteine (HCy), brain derived neurotrophic factor (BDNF), eicosapentaenoic acid (EPA), docosahexaenoic acid (DHA), omega-6/omega-3 FA ratio in serum; (iii) superoxide dismutase (SOD), glutathione peroxidase (GPx), and catalase (CAT) activities in erythrocyte hemolysate; and (iv) cortisol in saliva. The detailed methods, procedure descriptions, and results of the same cohort of patients are mentioned in our recent papers (7, 15, 21–23). For readers’ convenience, details of individual markers analyzes obtained from the same cohort of patients listed in **Table 1** are given in Supplements as Supplement - Methods.

### 2.7 Statistical analyses

StatsDirect^®^ 2.8.0 (StatsDirect Sales, Sale, Cheshire, M33 3UY, UK) and GraphPad Prism 9.5.1 (Insightful Science, California, USA) software were used for statistical processing and graphical display of data. We used mean ± standard deviation to describe normally distributed data. For data not normally distributed, we used the median, first and third quartile. We tested the normality of data with the Shapiro-Wilk test. To compare the control group and the group of depressed patients, we used the unpaired Student’s t-test (normally distributed data) or the Mann-Whitney U test if the data were not normally distributed. We tested the effect of supplementation using one-way ANOVA with repeated measures and Tukey’s *post-hoc* test or Friedman’s test with Dunn’s test. We used Spearman’s correlation coefficient to evaluate the dependence of two variables. For all statistical tests, a p < 0.05 was considered as statistically significant.

## 3 Results

### 3.1 Baseline data

Baseline characteristics of patients and healthy controls, such as age, weight, height, and BMI (kg/m2) were published previously (22), for readers’ convenience, the data are provided in Supplements as **Supplementary Table 1**. No differences in basic characteristics were found between the patients and the control group. However, in patients, but not in controls, significant differences were found between male and female in patient weight and height.

### 3.2 Metabolites of kynurenine and serotonin pathways in depressed patients and controls

Baseline levels of metabolites from the kynurenine and serotonin pathways, as well as NEO and BIO in urine are presented in **Table 1**. We observed significantly higher values of the KYN/TRP ratio in patients compared to healthy controls (p < 0.01). Additionally, TRP (p = 0.02) and its serotonin pathway metabolites—5-HTP (p < 0.01), SER (p = 0.02), and 5-HIAA (p = 0.02)—were significantly lower in patients than in controls (**Table 1**). At the baseline, no significant differences were found in the levels of NEO and BIO between patients and healthy controls. After dividing patients and healthy controls according to gender, we did not find significant differences between male and female in both groups of children and adolescents.

**Table 1:** Baseline levels of TRP catabolism metabolites as well as neopterin and biopterin in urine in depressed patients and healthy controls.

Metabolite [μmol/mmol CR]	Patients				Healthy Controls				P vs. C
	all	F	M	*p* F vs. M	all	F	M	*p* F vs. M	
	n=57	n=46	n=11		n=20	n=12	n=8		
TRP	3.16 (2.65; 4.12)	3.20 (2.65; 4.32)	3.15 (2.54; 3.92)	0.963 ^b^	4.41 (3.31; 7.16)	4.19 (2.86; 5.05)	6.63 (3.48; 9.40)	0.270 ^b^	**0.017 ^b^**
KYN/TRP	0.75 (0.60; 1.02)	0.76 (0.63; 0.91)	0.66 (0.48; 1.15)	0.699 ^b^	0.52 (0.37; 0.60)	0.49 (0.37; 0.63)	0.53 (0.36;0.56)	0.657 ^b^	**< 0.0001 ^b^**
KYN	2.48 (2.10; 3.01)	2.48 (2.10; 2.97)	2.64 (1.92; 3.13)	0.825 ^b^	2.52 (1.65; 3.53)	2.78 (1.12; 3.26)	3.2 (1.91; 3.59)	0.613 ^b^	0.944 ^b^
5-HTP	0.070 (0.054; 0.090)	0.070 (0.054; 0.094)	0.065 (0.046;0.090)	0.616 ^b^	0.116 (0.072; 0.264)	0.093 (0.072;0.173)	0.136 (0.087; 0.383)	0.263 ^b^	**0.003 ^b^**
SER	0.143 (0.081; 0.287)	0.151 (0.078; 0.315)	0.140 (0.113; 0.162)	0.792 ^b^	0.225 (0.151; 0.456)	0.209 (0.115; 0.513)	0.306 (0.184; 0.456)	0.473 ^b^	**0.021 ^b^**
5-HIAA	1.68 ± 0.37	1.72 ± 0.37	1.49 ± 0.27	0.062 ^a^	2.03 ± 0.60	2.04 ± 0.54	2.01 ± 0.71	0.909 ^a^	**0.023 ^a^**
NEO	0.19 (0.14; 0.27)	0.19 (0.15; 0.27)	0.14 (0.11; 0.19)	0.142 ^b^	0.20 (0.17; 0.29)	0.21 (0.17;0.29)	0.20 (0.14; 0.30)	0.689 ^b^	0.228 ^b^
BIO	0.40 ± 0.12	0.40 ± 0.13	0.35 ± 0.09	0.227 ^a^	0.42 ± 0.12	0.42 ± 0.10	0.43 ± 0.16	0.881 ^a^	0.414 ^a^

CR, creatinine; TRP, tryptophan; KYN, kynurenine; 5-HTP, 5-hydroxytryptophan; SER, serotonin; 5-HIAA, 5-hydroxyindole acetic acid; NEO, neopterin; BIO, biopterin; p, significance; F, female; M, male; P, patients; C, healthy controls; a, unpaired t-test; b, Man-Whitney U test.

Bold values indicate significant p value.

### 3.3 Effect of FA supplementation

The effect of omega-3 and omega-6 fatty acids on markers of the kynurenine pathway, as well as NEO and BIO, is presented in **Table 2**. Baseline TRP levels were not significantly different between the Om3 and Om6 groups. Administration of omega-3 FA did not affect the TRP level during the 12 weeks of supplementation. However, omega-6 FA administration significantly reduced TRP levels after 6 weeks and marginally significant after 12 weeks. Administration of the omega-3 FA emulsion induced an increased KYN/TRP ratio (representing IDO enzyme activity) and the level of KYN after 12 weeks of supplementation. Administration of omega-6-FA lead to an increase of the KYN/TRP ratio by week 6, but by week 12 the increase was marginally significant (p = 0.051). Comparison of metabolite levels in the Om3 and Om6 groups showed a significant difference in the KYN/TRP ratio by week 6, and in KYN at both the 6- and 12-week study points (**Table 2**).

**Table 2:** The effect of omega-3 and omega-6 FA on the metabolites of tryptophan, NEO and BIO in urine.

Metabolite [μmol/mmol CR]	Om3 (n = 27)					Om6 (n = 27)						*p* between Om3 and Om6		
	Week 0	Week 6	*p* between 0 and 6	Week 12	*p* between 0 and 12	Week 0	Week 6	*p* between 0 and 6	Week 12	*p* between 0 and 12	Week 0		Week 6	Week 12
TRP	3.28 ± 1.14	3.40 ± 1.28	0.832 ^b^	3.23 ± 1.01	0.964 ^b^	3.39 ± 1.04	3.03 ± 0.97	**0.050 ^b^**	3.07 ± 0.85	0.066 ^b^	0.712 ^c^		0.249 ^c^	0.514 ^c^
KYN/TRP	0.88 ± 0.32	1.00 ± 0.35	0.128 ^b^	1.06 ± 0.39	**0.045 ^b^**	0.72 ± 0.18	0.91 ± 0.30	**0.035 ^b^**	0.91 ± 0.34	0.051 ^b^	0.132 ^c^		**0.031 ^c^**	0.157 ^c^
KYN	2.65 ± 0.59	3.10 ± 0.72	**0.021 ^b^**	3.09 ± 0.77	**0.048 ^b^**	2.41 ± 0.61	2.58 ± 0.63	0.534 ^b^	2.48 ± 0.56	0.882 ^b^	0.137 ^c^		**0.007 ^c^**	**0.002 ^c^**
5-HTP	0.071 (0.056; 0.104)	0.080 (0.065; 0.144)	**0.008 ^a^**	0.082 (0.058; 0.108)	**0.035 ^a^**	0.070 (0.051; 0.090)	0.071 (0.053; 0.116)	0.742 ^a^	0.062 (0.053; 0.094)	0.999 ^a^	0.452 ^d^		0.144 ^d^	0.059 ^d^
SER	0.145 (0.097; 0.299)	0.167 (0.089; 0.311)	0.829 ^a^	0.149 (0.082; 0.298)	0.999 ^a^	0.094 (0.069; 0.266)	0.134 (0.099; 0.286)	0.353 ^a^	0.144 (0.096; 0.283)	0.999 ^a^	0.113 ^d^		0.847 ^d^	0.921 ^d^
5-HIAA	1.66 ± 0.41	1.54 ± 0.45	0.486 ^b^	1.52 ± 0.42	0.294 ^b^	1.69 ± 0.34	1.64 ± 0.38	0.886 ^b^	1.60 ± 0.42	0.541 ^b^	0.722 ^c^		0.390 ^c^	0.553 ^c^
NEO	0.19 (0.13; 0.27)	0.19 (0.15; 0.25)	0.999 ^a^	0.19 (0.13; 0.27)	0.995 ^a^	0.19 (0.14; 0.30)	0.19 (0.16; 0.25)	0.999 ^a^	0.19 (0.15; 0.29)	0.999 ^a^	0.727 ^d^		0.870 ^d^	0.669 ^d^
BIO	0.37 ± 0.12	0.41 ± 0.12	0.239 ^b^	0.44 ± 0.17	**0.023 ^b^**	0.41 ± 0.10	0.42 ± 0.12	0.835 ^b^	0.43 ± 0.12	0.687 ^b^	0.236 ^c^		0.758 ^c^	0.742 ^c^

Om3, omega-3 group; Om6, omega-6 group; CR, creatinine; TRP, tryptophan; KYN, kynurenine; 5-HTP, 5 -hydroxytryptophan; SER, serotonin; 5-HIAA, 5-hydroxyindole acetic acid; NEO, neopterin; BIO, biopterin; p, significance; a, Friedman test with Dunn’s post hoc test; b, Repeated-measures one-way ANOVA with Tukey’s post hoc test; c, unpaired t-test; d, Man-Whitney U test.

Bold values indicate significant p value.

Serotonin pathway metabolites were significantly influenced only by omega-3 FA. The first metabolite, 5-HTP, shoved a slight elevation at week 6 (13%) and week 12 (15%). However, no differences between the Om3 and Om6 groups were observed in individual weeks (**Table 2**).

The level of NEO was not affected by the supplementation of either omega-3 or omega-6 FAs. However, the BIO level increased after 12 weeks (19%) of omega-3 FA supplementation (p=0.023), but not with omega-6 FA supplementation (**Table 2**).

### 3.4 Correlations between metabolites of tryptophan catabolic pathways, as well as neopterin and biopterin, and markers of oxidative stress and other markers related to depressive disorder

We investigated the association between metabolites of TRP catabolism, NEO, and BIO, and oxidative stress markers (8-isoP, LP, AOPP, NT, TEAC, SOD, GPx, CAT), as detailed in our published paper from the same patient cohort (22). Additionally, we explored correlations with inflammation markers (TXB, HCy), BDNF (23), lipid profile markers (21), EPA, DHA, omega-6/omega-3 FA ratio, CDI score (7), and salivary cortisol (15) (refer to **Table 3**). Only significant and marginally significant correlations are presented in **Table 3**, as other markers did not exhibit a notable correlation.

**Table 3:** Significant and marginally significant correlations between metabolites of TRP catabolism and markers of oxidative stress and selected parameters related to the pathophysiology of depressive disorder at baseline.

All Patients at baseline	n	r	*p*
5-HTP and LP	50	0.259	*0.069*
SER and LP	50	0.285	**0.045**
SER and AOPP	57	0.301	**0.023**
SER and NT	57	−0.393	**0.039**
5-HIAA and BDNF	50	−0.284	**0.045**
5-HIAA and GPx	57	−0.336	**0.011**
5-HIAA and Chol	57	0.335	**0.011**
5-HIAA and Cortisol	55	0.262	*0.051*
KYN/TRP and Cortisol	55	0.268	**0.048**
KYN/TRP and Om6/Om3	53	0.292	**0.034**
KYN and TEAC	57	0.352	**0.007**
NEO vs. GPx	57	-0.275	**0.038**
BIO vs. TXB	53	-0.311	**0.024**
5-HIAA and CDI	57	0.334	**0.011**

TRP, tryptophan; KYN, kynurenine; 5-HTP, 5 -hydroxytryptophan; SER, serotonin; 5-HIAA, 5-hydroxyindoleacetic acid; NEO, neopterin; BIO, biopterin; LP, lipoperoxides; AOPP, advanced oxidation protein products; NT, nitrotyrosine; GPx, glutathione peroxidase; TEAC, trolox equivalent antioxidant capacity; BDNF, brain derived neutrophic factor; Chol, total cholesterol; Om3, omega-3 fatty acids; Om6, omega-6 fatty acids; CDI, Children’s Depression Inventory; n, group size; r, Spearman’s rank correlation coefficient; p, significance.

Bold values indicate significant p value.

In terms of the correlations between the severity of depression, as measured by CDI scores, and the studied metabolites at baseline, only the correlation of CDI with 5-HIAA was found to be significant (r=0.334, p=0.011, n=57).

## 4 Discussion

In this paper, we focused on tracking the associations between TRP metabolites, pteridines (neopterin, biopterin), and selected markers from the same cohort of depressive children and adolescents already published [markers of lipid profiles and oxidative stress (21, 22), markers of inflammation response (23), omega-6/omega-3 FA ratio, and CDI score (7), and cortisol (15)].

The molecular mechanisms of depressive and anxiety disorders in children are not completely known (5, 6, 25). In our group of patients with depressive disorder, we found a lower level of TRP in urine compared to controls (**Table 1**). Both, increased (19, 26) and decreased (27, 28) TRP levels as well as heterogeneous results of KYN and KYP/TRP ratio (19, 26) were found in adult patients. Savitz et al. (29) reported unaltered levels of TRP metabolites in the kynurenine pathway in adult patients with depressive disorder and patients in remission compared to healthy controls.

Only a few results on tryptophan catabolism in children and adolescents with depressive disorder are available. DeWitt et al. (25) found no difference in the KYN/TRP ratio and kynurenic acid between depressed adolescents and healthy controls. In our study of depressed children and adolescents, we found decreased urinary TRP levels (**Table 1**). However, the KYN/TRP ratio, by Öztürk et al. (8), was higher compared to the controls (**Table 1**). The redirection of TRP metabolism to the kynurenine pathway rather than to the serotonin pathway in our cohort of patients is supported by the negative correlation between KYN/TRP and SER (r = − 0.273, p = 0.041). The levels of KYN were not significantly different between depressive patients and healthy controls in our study (**Table 1**) as well as in the study of Öztürk et al. (8). As we did not determine kynurenic acid (KA) (neuroprotective metabolite) for technical reasons, nor quinolinic acid (QUIN) (neurotoxic metabolite), we cannot further elaborate on the latter finding.

**Figure 3:**
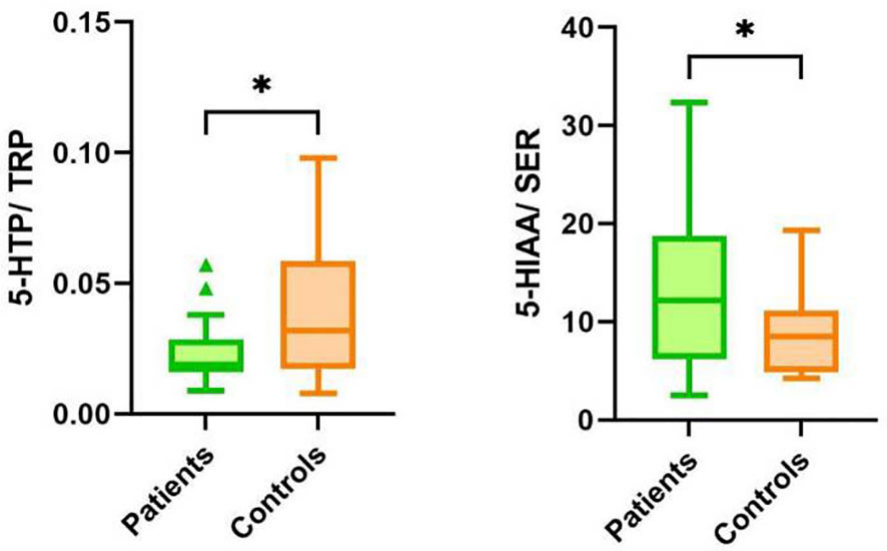
Ratios of metabolites in the serotonin pathway of TRP catabolism in patients and controls. TRP, tryptophan; 5-HTP, 5-hydroxytryptophan; SER, serotonin; 5-HIAA, 5-hydroxyindoleacetic acid, *p < 0.05.

The second pathway of TRP catabolism is the serotonin pathway (**Figure 1**). In our patients, the levels of 5-HTP, SER, and 5-HIAA were significantly lower compared to healthy controls. Since baseline TRP levels in patients and healthy controls differed, we recalculated the ratios of individual metabolites of the serotonin pathway. The 5-HTP/TRP ratio was significantly lower in patients (p = 0.017) (**Figure 3**), supporting our previous findings of redirecting TRP catabolism to the kynurenine pathway. The SER/5-HTP ratio was not different between patients and controls, while the 5-HIAA/SER ratio was significantly higher in patients compared to controls (p = 0.031) (**Figure 3**). This suggests that monoamine oxidase (MAO) activity (represented by the 5-HIAA/SER ratio) was increased in patients, leading to the increased formation of 5-HIAA. Similarly, Jones and Raghanti (30) found increased activity and expression of MAO in both depressed patients and animal experiments. This finding is consistent with the study of Jayamohanan et al. (10), who found that the level of 5-HIAA increases with stress. In our pediatric patients, the level of 5-HIAA marginally significantly positively correlates with cortisol level (p=0.051), significantly with the severity of depression (CDI score) (p=0.011), and negatively with brain-derived neurotrophic factor (BDNF) (p=0.045). These findings point to the involvement of 5-HIAA in the pathophysiology of depressive disorder. The low levels of SER and 5-HIAA in CSF are associated with suicide attempts (16, 31). A low cholesterol level in adult patients is also correlated with suicide attempts (32). Similarly, we also found a positive correlation between total cholesterol (21) and 5-HIAA in the urine of our pediatric patients (**Table 3**). Patients with depressive disorder in our study did not report suicidal attempts, which are associated with low 5-HIAA and cholesterol levels (16, 32).

Omega-3 fatty acids, EPA, DHA, and their ratio play an important role in the pathophysiology of depressive disorders (7). In a recent review, Zhou et al. (33) discussed an antidepressant effect of omega-3 FA. Carabelli et al. (34) found that fish oil high in omega-3 fatty acids inhibited indoleamine 2,3-dioxygenase (IDO) and increased SER in the male Wistar rat model system after induction of depression with lipopolysaccharide. In another model system, omega-3 fatty acids positively influenced the reduction of neurogenesis caused by pro-inflammatory cytokines by increasing neurogenesis. Moreover, EPA and DHA inhibited the formation of KYN, QUIN, and mRNA expression of the enzyme kynurenine-3-monooxygenase (35). Fish oil supplementation has been reported to decrease the expression of IDO (represents KYN/TRP ratio) and increase the level of SER in the hippocampus (34).

We found that omega-3-FA supplementation increases the urinary KYN/TRP ratio (representing IDO activity) after 12 weeks of supplementation and increases KYN levels after 6 and 12 weeks of omega-3-FA supplementation. At the same time, omega-3 FAs increased the level of the first metabolite of the serotonin pathway, 5-HTP, after 6 and 12 weeks (**Table 2**).

Similarly, omega-6 FA increases the KYN/TRP ratio after 6 weeks of supplementation, but after 12 weeks the increase is borderline significant. The serotonin pathway is not affected by omega-6 FAs.

The stimulatory effect of omega-3 FA on the formation of KYN found in our work contradicts the findings of Carabelli et al. (34) and Borsini et al. (35). As we mentioned above, we did not determine either KA or QUIN and therefore cannot assess whether the transformation of KYN was more neuroprotective (through KA formation) or neurotoxic (through the formation of QUIN). This would help to explain the relationship between omega-3 FAs and their possible positive or negative effect on the kynurenine pathway of TRP catabolism. We do not assume that the increased level of KYN after omega-3 FA supplementation would have a fundamental effect on the severity of depression, as the correlations between the CDI score and KYN (r=−0.055, p=0.683), or the KYN/TRP ratio (r=−0.074, p=0.585) were not significant at baseline or after 6 or 12 weeks of omega-3 FA supplementation.

Omega-3-FA supplementation increased 5-HTP by 15% after 12 weeks. SER was increased non-significantly by 15.2% after 6 weeks. We can only speculate that omega-3 FA exerts their antidepressant effect, except for other mechanisms (7), through the weak stimulation of the serotonin pathway of TRP catabolism rather than through inhibition of the kynurenine pathway.

No correlation was found between the studied metabolites of TRP degradation and the concentrations of EPA and DHA FAs determined in the same cohort of patients (7). However, in the present study, it was found that the omega-6/omega-3 FA ratio positively correlates with KYN/TRP (r = 0.292, p = 0.034) in patients. These findings align with the conclusions of Oxenkrug (11), who discovered that omega-3 FAs transcriptionally inhibit IDO expression. Trebatická et al. (7) also found that the severity of depressive disorder in children and adolescents is more influenced by the ratio of omega-6 to omega-3 fatty acids than by the levels of EPA and DHA alone. It can be assumed that the current correlation between the omega-6 to omega-3 fatty acid ratio and the KYN/TRP ratio supports the importance of fatty acid composition in the pathophysiology of depressive disorder. The positive effect of omega-3 FA can also be attributed to their anti-inflammatory activity (22, 33) (**Figure 1**).

The metabolic pathways of tryptophan (TRP) breakdown are significantly affected by high levels of permanent stress. Stress activates the HPA axis, leading to excessive cortisol production. Cortisol, in turn, activates the IDO/TDO enzymes and the kynurenine pathway through the production of pro-inflammatory cytokines (**Figure 1**). In a study by Michels et al. (14), the relationship between TRP catabolism, psychological stress, and inflammation was evaluated. The authors concluded that psychological stress in adolescents correlated with kynurenine pathway metabolites in the presence of high inflammation. In our recent study (15), we measured the level of salivary cortisol in depressed patients. However, direct markers of the inflammatory response were not monitored, and high-sensitivity C-reactive protein (hsCRP) did not indicate strong inflammation. Midday cortisol showed a significant but weak correlation between cortisol and the KYN/TRP ratio (r = 0.268, p = 0.048) and a borderline significant correlation with the final metabolite of the serotonin pathway, 5-HIAA (r = 0.262, p = 0.051). Our results suggest that in children and adolescents with depressive disorder, stress represented by cortisol can initiate the kynurenine pathway and possibly activate monoamine oxidase (MAO), leading to the subsequent decomposition of serotonin (SER) into 5-HIAA and potentially contributing to the worsening of depression.

Metabolites of the kynurenine pathway, 3-HK, and QUIN, are known pro-oxidative metabolites, forming superoxide, hydroxyl radical, and hydrogen peroxide (36). The superoxide can react with NO to form the highly reactive peroxynitrite, ONOO−. The activation of the kynurenine pathway contributes to the formation of reactive oxygen and nitrogen species and thereby increases oxidative stress (11). The oxidation of SER to 5-HIAA in the serotonin pathway catalyzed by MAO also produces the pro-oxidant, H_2_O_2_ (37). The onset of oxidative stress goes hand in hand with the activation of inflammation, leading to the activation of IDO and thus kynurenine catabolism (38).

We presented the association of oxidative stress with depressive disorder in children and adolescents in a recent paper (22). We found an increased level of 8-isoprostanes in urine (later products of lipoperoxidation), AOPP, and NT, and reduced activity of GPx compared to healthy children. We also found a strong positive correlation of depression severity, CDI with NT at baseline, and a negative correlation with TEAC, SOD, and GPx. In this study, we correlated oxidative stress markers (22) with metabolites of the kynurenine and serotonin pathways. Surprisingly, neither KYN/TRP nor KYN correlated with markers of oxidative stress. However, SER positively correlated with LP and AOPP. This could be attributed to the fact that in depression, the activity of MAO (the enzyme that catalyzes the conversion of SER to 5-HIAA) (**Figure 1**) is increased and that H_2_O_2_ formed during the catalytical reaction can trigger lipoperoxidation and oxidative damage to proteins (39). Reduced GPx activity also contributes to the formation of LP (a marker of the earlier phase of lipoperoxidation) (22). These connections are also supported by our present finding of a negative correlation between 5-HIAA and GPx in depressive patients (**Table 3**).

In our recent work (22), we posited that the causal marker of oxidative stress in adolescents with depressive disorder is NT. Nobis et al. (40) found a decreased level of TRP and an increased level of NT in the urine of adult depressive patients by our results in children and adolescents. This supports our assertion that nitrosative stress also plays a role in the pathophysiology of depressive disorder (41). Notably, in the present work, NT negatively correlated with SER (r =−0.393, p=0.039) in patients with depressive disorder, supporting our previous observation.

The observed negative correlation between 5-HIAA and BDNF in our pediatric patients (**Table 3**) leads us to speculate that 5-HIAA may negatively affect the maintenance and survival of neurons and synaptic plasticity regulated by BDNF (41). The kynurenine and serotonin pathway metabolites did not correlate with CDI, except for the correlation between 5-HIAA and CDI in depressed patients (r = 0.334, p = 0.011). This result, along with the negative correlation between 5-HIAA and BDNF, hints at the importance of 5-HIAA in the pathophysiology of depressive disorder in children and adolescents.

Information on NEO and BIO levels in depressed children and adolescents is only sporadic and quite heterogeneous. In our pediatric and adolescent patients, we did not detect a difference in NEO and BIO from healthy controls. Deac et al. (2016) found in healthy non-inflamed young individuals (18-28 y.) a positive correlation of NEO with KYN and KYN/TRP ratio and a negative correlation with TRP (42). In our work, we did not find a significant correlation between NEO, BIO, and metabolites of TRP catabolism neither in healthy children nor in depressed patients. However, we found in depressed patients a negative correlation between NEO and the antioxidant enzyme GPx (22), between BIO and the inflammatory marker TXB (23), and a positive correlation between BIO and total antioxidant capacity (TEAC) (22). These findings confirm the involvement of inflammation, oxidative stress, and imbalance in TRP catabolism pathways in the pathophysiology of depressive disorder in children and adolescents.

The level of NEO in the urine of depressed patients was not affected by either omega-3 or omega-6 FA during the 12-week supplementation period. However, BIO increased significantly after 12 weeks of omega-3 FA but not omega-6 FA supplementation. There are no known studies on the relationship between NEO, omega-3 FA, and depression. However, in adult patients with coronary artery disease, a weak negative correlation was found between fish intake and KYN/TRP ratio and omega-3 FA intake and plasma NEO (43). Since there is not enough information about the role of BIO and tetrahydrobiopterin in the pathophysiology of depressive disorder, this topic is currently a matter of discussion.

The presented study has certain limitations. Gender differences were not thoroughly explored due to the small number of boys included in the study. This is attributed to the higher prevalence of depressive disorder in girls and the general reluctance of boys to provide biological samples. Additionally, the patient group consisted of a maximum of 58 individuals (46 females and 12 males), while the healthy control group comprised from ethical reason only 20 individuals (12 females and 8 males). Nutritional habits of patients and controls were not monitored, preventing a complete explanation for the baseline difference in urinary TRP levels between patients and controls. However, participants were instructed to follow a standard diet with an obligation to inform the responsible doctor about any deviations in eating habits. Furthermore, the study did not measure the levels of quinolinic acid (QUIN) or kynurenic acid (KA), which would offer a more detailed understanding of the balance in TRP metabolism (neurotoxic versus neuroprotective pathways) and the involvement of TRP metabolites in the pathophysiology of depressive disorder.

## 5 Conclusion

The impact of both omega-3 fatty acids (FAs) and omega-6 FAs was evident in the increased activation of the KYN/TRP ratio. The lack of QUIN and KA measurements limits our ability to explain these findings and assess the potential influence of fatty acids on the degradation of TRP concerning the neurotoxic or neuroprotective functions of its metabolites. Interestingly, omega-3 FAs, but not omega-6 FAs, led to an increase in the production of the first metabolite of the serotonin pathway, 5-HTP. We established a connection between the kynurenine and serotonin pathways of TRP catabolism and depressive disorder in children and adolescents.

However, no association was observed between NEO and BIO and the metabolites of TRP degradation. While cortisol was found to stimulate the kynurenine pathway of TRP catabolism, it showed no correlation with the severity of depression. On the other hand, the terminal metabolite of the serotonin pathway, 5-HIAA, exhibited a significant positive correlation with CDI and a negative correlation with BDNF. This supports the idea that 5-HIAA plays a role in the pathophysiology of depressive disorder in children and adolescents.

We confirmed the link between oxidative stress and the catabolism of TRP in depressive children. NEO shows an association with oxidative stress through a negative correlation with glutathione peroxidase (GPx), and biopterin (BIO) shows a negative correlation with TXB.

Importantly, we were the first to underscore the significance of the precise omega-6/omega-3 fatty acid ratio and its correlation with the kynurenine pathway of TRP degradation (KYN/TRP ratio) in the pathophysiology of depressive disorders in children and adolescents.

It’s crucial to note that depressive disorder is a highly multifactorial condition. Therefore, the generalization of our findings necessitates further studies with precise diagnoses and larger study cohorts.

## Data availability statement

The original contributions presented in the study are included in the article/**Supplementary Material**. Further inquiries can be directed to the corresponding author.

## Ethics statement

The studies involving humans were approved by Ethics Committee of the National Institute of Children’s diseases and the Faculty of Medicine, Comenius University Bratislava (20 March 2013). The studies were conducted in accordance with the local legislation and institutional requirements. Written informed consent for participation in this study was provided by the participants’ legal guardians/next of kin. Children provided verbal assent before participation in the study.

## Author contributions

LI: Formal analysis, Visualization, Writing – original draft. MM: Formal analysis, Writing – review & editing. JM: Methodology, Writing – review & editing. ZP: Formal analysis, Writing – review & editing. IG: Methodology, Writing – review & editing. ZĎ: Conceptualization, Project administration, Supervision, Writing – review & editing, Funding acquisition. LŠ: Conceptualization, Supervision, Visualization, Writing – review & editing. JT: Conceptualization, Investigation, Supervision, Writing – review & editing.

## References

[1] Costello EJ Erkanli A Angold A. Is there an epidemic of child or adolescent depression? J Child Psychol Psychiatry (2006) 47:1263–71. doi: 10.1111/j.1469-7610.2006.01682.x 17176381

[2] Lim GY Tam WW Lu Y Ho CS Zhang MW Ho RC. Prevalence of depression in the community from 30 countries between 1994 and 2014. Sci Rep (2018) 8:2861. doi: 10.1038/s41598-018-21243-x 29434331 PMC5809481

[3] Coghill D Bonnar S Seth S Duke S Graham J. Child and Adolescent Psychiatry. 1st ed. New York, USA: Oxford University Press (2009). p. 491.

[4] Maes M Leonard BE Myint AM Kubera M Verkerk R. The new ‘5-HT’ hypothesis of depression: Cell-mediated immune activation induces indoleamine 2,3-dioxygenase, which leads to lower plasma tryptophan and an increased synthesis of detrimental tryptophan catabolites (TRYCATs), both of which contribute to the onset of depression. Prog Neuropsychopharmacol Biol Psychiatry (2011) 35:702–21. doi: 10.1016/j.pnpbp.2010.12.017 21185346

[5] Vaváková M Ďuračková Z Trebatická J. Markers of oxidative stress and neuroprogression in depression disorder. Oxid Med Cell Longev (2015) 2015:898393. doi: 10.1155/2015/898393 26078821 PMC4453280

[6] Cernackova A Durackova Z Trebaticka J Mravec B. Neuroinflammation and depressive disorder: The role of the hypothalamus. J Clin Neurosci (2020) 75:5–10. doi: 10.1016/j.jocn.2020.03.005 32217047

[7] Trebatická J Hradečná Z Surovcová A Katrenčíková B Gushina I Waczulíková I. Omega-3 fatty-acids modulate symptoms of depressive disorder, serum levels of omega-3 fatty acids and omega-6/omega-3 ratio in children. A randomized, double-blind and controlled trial. Psychiatry Res (2020) 287:112911. doi: 10.1016/J.PSYCHRES.2020.112911 32179212

[8] Öztürk M Yalın Sapmaz Ş Kandemir H Taneli F Aydemir Ö. The role of the kynurenine pathway and quinolinic acid in adolescent major depressive disorder. Int J Clin Pract (2021) 75:e13739. doi: 10.1111/IJCP.13739 32997876

[9] Guillemin GJ. Quinolinic acid, the inescapable neurotoxin. FEBS J (2012) 279:1356–65. doi: 10.1111/J.1742-4658.2012.08485.X 22248144

[10] Jayamohanan H Kumar MKM Aneesh TP. 5-HIAA as a potential biological marker for neurological and psychiatric disorders. Adv Pharm Bull (2019) 9:374–81. doi: 10.15171/APB.2019.044 PMC6773935 31592064

[11] Oxenkrug G. Serotonin – kynurenine hypothesis of depression: historical overview and recent developments. Curr Drug Targets (2013) 14:514–21. doi: 10.2174/1389450111314050002 PMC3726541 23514379

[12] Higuchi Y Soga T Parhar IS. Regulatory pathways of monoamine oxidase A during social stress. Front Neurosci (2017) 11:604. doi: 10.3389/FNINS.2017.00604 29163009 PMC5671571

[13] Ďuračková Z. Some current insights into oxidative stress. Physiol Res (2010) 59:459–69. doi: 10.33549/PHYSIOLRES.931844 19929132

[14] Michels N Clarke G Olavarria-Ramirez L Gómez-Martínez S Díaz LE Marcos A. Psychosocial stress and inflammation driving tryptophan breakdown in children and adolescents: A cross-sectional analysis of two cohorts. Psychoneuroendocrinology (2018) 94:104–11. doi: 10.1016/j.psyneuen.2018.05.013 29775873

[15] Oravcova H Katrencikova B Garaiova I Durackova Z Trebaticka J Jezova D. Stress hormones cortisol and aldosterone, and selected markers of oxidative stress in response to long-term supplementation with omega-3 fatty acids in adolescent children with depression. Antioxidants (2022) 11:1546. doi: 10.3390/ANTIOX11081546 36009265 PMC9405235

[16] Correia AS Vale N. Tryptophan metabolism in depression: A narrative review with a focus on serotonin and kynurenine pathways. Int J Mol Sci (2022) 15:987697. doi: 10.3390/IJMS23158493 PMC9369076 35955633

[17] Erhardt S Olsson SK Engberg G. Pharmacological manipulation of kynurenic acid. CNS Drugs (2009) 23:91–101. doi: 10.2165/00023210-200923020-00001 19173370

[18] Parrott JM O’Connor JC. Kynurenine 3-monooxygenase: An influential mediator of neuropathology. Front Psychiatry (2015) 6:116/BIBTEX. doi: 10.3389/FPSYT.2015.00116/BIBTEX 26347662 PMC4542134

[19] Almulla AF Maes M. The tryptophan catabolite or kynurenine pathway’s role in major depression. Curr Top Med Chem (2022) 22:1731–5. doi: 10.2174/1568026622666220428095250 36321312

[20] Cavaleri D Bartoli F Capogrosso CA Guzzi P Moretti F Riboldi I. Blood concentrations of neopterin and biopterin in subjects with depression: A systematic review and meta-analysis. Prog Neuropsychopharmacol Biol Psychiatry (2023) 120:110633. doi: 10.1016/j.pnpbp.2022.110633 36089162

[21] Katrenčíková B Vaváková M Waczulíková I Oravec S Garaiova I Nagyová Z. Lipid profile, lipoprotein subfractions, and fluidity of membranes in children and adolescents with depressive disorder: effect of omega-3 fatty acids in a double-blind randomized controlled study. Biomolecules (2020) 10:1427. doi: 10.3390/BIOM10101427 33050072 PMC7650679

[22] Katrenčíková B Vaváková M Paduchová Z Nagyová Z Garaiova I Muchová J. Oxidative stress markers and antioxidant enzymes in children and adolescents with depressive disorder and impact of omega-3 fatty acids in randomised clinical trial. Antioxidants (2021) 10:1256. doi: 10.3390/ANTIOX10081256 34439504 PMC8389273

[23] Paduchová Z Katrenčíková B Vaváková M Laubertová L Nagyová Z Garaiova I. The effect of omega-3 fatty acids on thromboxane, brain-derived neurotrophic factor, homocysteine, and vitamin d in depressive children and adolescents: Randomized controlled trial. Nutrients (2021) 13:1095. doi: 10.3390/NU13041095 33801688 PMC8066966

[24] Skorobogatov K De Picker L Verkerk R Coppens V Leboyer M Müller N. Brain versus blood: A systematic review on the concordance between peripheral and central kynurenine pathway measures in psychiatric disorders. Front Immunol (2021) 12:716980. doi: 10.3389/FIMMU.2021.716980 34630391 PMC8495160

[25] DeWitt SJ Bradley KA Lin N Yu C Gabbay V. A pilot resting-state functional connectivity study of the kynurenine pathway in adolescents with depression and healthy controls. J Affect Disord (2018) 227:752–8. doi: 10.1016/j.jad.2017.11.040 PMC5805652 29254065

[26] Pompili M Lionetto L Curto M Forte A Erbuto D Montebovi F. Tryptophan and kynurenine metabolites: are they related to depression? Neuropsychobiology (2019) 77:23–8. doi: 10.1159/000491604 30110684

[27] Maes M Meltzer HY Scharpè S Bosmans E Suy E De Meester I. Relationships between lower plasma L-tryptophan levels and immune-inflammatory variables in depression. Psychiatry Res (1993) 49:151–65. doi: 10.1016/0165-1781(93)90102-M 7908745

[28] Miura H Ozaki N Sawada M Isobe K Ohta T Nagatsu T. A link between stress and depression: shifts in the balance between the kynurenine and serotonin pathways of tryptophan metabolism and the etiology and pathophysiology of depression. Stress (2008) 11:198–209. doi: 10.1080/10253890701754068 18465467

[29] Savitz J Drevets WC Wurfel BE Ford BN Bellgowan PSF Victor TA. Reduction of kynurenic acid to quinolinic acid ratio in both the depressed and remitted phases of major depressive disorder. Brain Behav Immun (2015) 46:55–9. doi: 10.1016/j.bbi.2015.02.007 PMC4414807 25686798

[30] Jones DN Raghanti MA. The role of monoamine oxidase enzymes in the pathophysiology of neurological disorders. J Chem Neuroanat (2021) 114:101957. doi: 10.1016/J.JCHEMNEU.2021.101957 33836221

[31] Placidi GPA Oquendo MA Malone KM Huang YY Ellis SP Mann JJ. Aggressivity, suicide attempts, and depression: relationship to cerebrospinal fluid monoamine metabolite levels. Biol Psychiatry (2001) 50:783–91. doi: 10.1016/S0006-3223(01)01170-2 11720697

[32] Lester D. Serum cholesterol levels and suicide: A meta-analysis. Suicide Life-Threatening Behav (2002) 32:333–46. doi: 10.1521/SULI.32.3.333.22177 12374479

[33] Zhou L Xiong JY Chai YQ Huang L Tang ZY Zhang XF. Possible antidepressant mechanisms of omega-3 polyunsaturated fatty acids acting on the central nervous system. Front Psychiatry (2022) 13:933704. doi: 10.3389/FPSYT.2022.933704 36117650 PMC9473681

[34] Carabelli B Delattre AM Waltrick APF Araújo G Suchecki D MaChado RB. Fish-oil supplementation decreases Indoleamine-2,3-Dioxygenase expression and increases hippocampal serotonin levels in the LPS depression model. Behav Brain Res (2020) 390:112675. doi: 10.1016/J.BBR.2020.112675 32407816

[35] Borsini A Alboni S Horowitz MA Tojo LM Cannazza G Su KP. Rescue of IL-1β-induced reduction of human neurogenesis by omega-3 fatty acids and antidepressants. Brain Behav Immun (2017) 65:230–8. doi: 10.1016/J.BBI.2017.05.006 PMC5540223 28529072

[36] Wang Q Liu D Song P Zou MH. Tryptophan-kynurenine pathway is dysregulated in inflammation, and immune activation. Front Biosci (Landmark Ed (2015) 20:1116–43. doi: 10.2741/4363 PMC4911177 25961549

[37] Kaludercic N Takimoto E Nagayama T Feng N Lai EW Bedja D. Monoamine oxidase A-mediated enhanced catabolism of norepinephrine contributes to adverse remodeling and pump failure in hearts with pressure overload. Circ Res (2010) 106:193–202. doi: 10.1161/CIRCRESAHA.109.198366 19910579 PMC2804073

[38] González Esquivel D Ramírez-Ortega D Pineda B Castro N Ríos C Pérez de la Cruz V. Kynurenine pathway metabolites and enzymes involved in redox reactions. Neuropharmacology (2017) 112:331–45. doi: 10.1016/J.NEUROPHARM.2016.03.013 26970015

[39] Khanzode SSDSSD Dakhale GN Khanzode SSDSSD Saoji A Palasodkar R. Oxidative damage and major depression: the potential antioxidant action of selective serotonin re-uptake inhibitors. Redox Rep (2003) 8:365–70. doi: 10.1179/135100003225003393 14980069

[40] Nobis A Zalewski D Samaryn E Maciejczyk M Zalewska A Waszkiewicz N. Urine 3-nitrotyrosine and serum HDL as potential biomarkers of depression. J Clin Med (2023) 12:377. doi: 10.3390/JCM12010377 36615177 PMC9821220

[41] Zhang J Yao W Hashimoto K. Brain-derived neurotrophic factor (BDNF)-trkB signaling in inflammation-related depression and potential therapeutic targets. Curr Neuropharmacol (2016) 14:721–31. doi: 10.2174/1570159X14666160119094646 PMC5050398 26786147

[42] Deac OM Mills JL Gardiner CM Shane B Quinn L Midttun Ø. Serum immune system biomarkers neopterin and interleukin-10 are strongly related to tryptophan metabolism in healthy young adults. J Nutr (2016) 146:1801–6. doi: 10.3945/jn.116.230698 PMC4997280 27489009

[43] Karlsson T Strand E Dierkes J Drevon CA Øyen J Midttun Ø. Associations between intake of fish and n-3 long-chain polyunsaturated fatty acids and plasma metabolites related to the kynurenine pathway in patients with coronary artery disease. Eur J Nutr (2017) 56:261–72. doi: 10.1007/s00394-015-1077-9 26482150

