# Supplementary material for: The kynurenine and serotonin pathway, neopterin and biopterin in depressed children and adolescents: an impact of omega-3 fatty acids, and association with markers related to depressive disorder. A randomized, blinded, prospective study
Source: Front Psychiatry. 2024 Feb 13;15:1347178. doi: 10.3389/fpsyt.2024.1347178 (PMC10896889; doi:10.3389/fpsyt.2024.1347178)
Supplement: Supplementary file 1 [file DataSheet_1.pdf]

Table S1. Baseline characteristics of depressed patients and healthy controls.

Parameter	Patients			*p*-Value	Healthy Controls			*p*-Value	*p*-Value
	All	Male	Female	M vs. F	All	Male	Female	M vs. F	P vs. C
n	58	12	46		20	8	12		
Age (years)	15.6 ± 1.6	16.4 ± 2.2	15.3 ± 1.3	0.235	14.8 ± 2.4	14.0 ± 2.5	14.4 ± 2.5	0.621	0.059
Weight (kg)	60.3 ± 11.7	68.2 ± 15.5	56.8 ± 9.3	**0.015**	54.9 ± 18.8	56.1 ± 21.2	54.1 ± 17.8	0.435	0.147
Height (m)	1.68 ± 0.1	1.74 ± 01	1.66 ± 0.1	**0.016**	1.6 ± 0.2	1.6 ± 0.2	1.6 ± 0.1	0.543	0.471
BMI (kg/m2)	21.14 ± 2.7	22.4 ± 3.6	20.5 ± 2.8	0.532	20.6 ± 4.2	20.1 ± 3.0	20.9 ± 5.0	0.498	0.537

M – male, F – female, P – patient, C – healthy controls, *p* – statistical significance, vs. – versus, n – number of individuals, *p* – values were analysed by a Man-Whitney U test.
