# Supplementary material for: The kynurenine and serotonin pathway, neopterin and biopterin in depressed children and adolescents: an impact of omega-3 fatty acids, and association with markers related to depressive disorder. A randomized, blinded, prospective study
Source: Front Psychiatry. 2024 Feb 13;15:1347178. doi: 10.3389/fpsyt.2024.1347178 (PMC10896889; doi:10.3389/fpsyt.2024.1347178)
Supplement: Supplementary file 2 [file DataSheet_2.pdf]

Supplement – Methods

Methods for determining the markers listed in Table 3 are given in this supplement.

1. **Determination of fatty acids (FA) in serum**

Venous blood samples were collected after 12-hour overnight fast. Within 1 h of collection, blood was centrifuged (1200 x g. 10 min), serum and plasma (EDTA as an anticoagulant) were obtained and frozen at −80 °C until analysis. Lipids were extracted using the method of Bligh and Dyer (1959). Fatty acid methyl esters (FAME) were prepared by transmethylation with 2.5% H_2SO_4 in dry methanol/toluene (2:1. by vol.). FAME was separated using a Clarus 500 gas chromatograph (Perkin-Elmer, Norwalk, Connecticut) fitted with a 30 m x 0.25 mm i.d. capillary column (Elite 225. Perkin Elmer). The oven temperature was programmed: 170 °C for 3 min. heated to 220 °C at 4 °C/min. held at 220 °C for 15 min. FAME was identified routinely by comparing retention times with fatty acid standards (Nu-Chek Prep. Inc., Elysian, USA). Fatty acid C17:0 was used as an internal standard. The concentration of FA was expressed as (μg/0.1 mL) (7).

2. **Determination of lipid profile**

The fasting serum levels of total cholesterol (TCH), LDL cholesterol, HDL cholesterol, triacylglycerols and creatinine in urine were determined at the Department of Clinical Biochemistry of National Institute of Children's Diseases using a Hitachi 911 Analyzer by a standard procedure using Roche Diagnostics kits to determine the individual parameters (Burgess Hill, RH159RY, UK) (21).

3. **Determination of thromboxane (TXB) and brain-derived neurotrophic factor (BDNF)**

Thromboxane B2 in plasma was determined with a Thromboxane B2 EIA kit (Cayman Chemicals, No. 501020, Ann Arbor, Michigan 48108, MI, USA), according to the manufacturer's protocol. The concentration of thromboxane is presented in pg/mL.
BDNF was determined in plasma with a Human BDNF ELISA kit (Sigma-Aldrich, No. RAB0026, St. Louis, MO, USA), according to the manufacturer's protocol, and is expressed in ng/mL (23).

4. **Determination of oxidative stress markers**

The concentration of lipoperoxides (LP) was determined in the serum according to El-Saadani et al. The determination was based on the ability of peroxides to convert oxidatively iodine (I^-) to iodine (I_2). The iodine in the reaction mixture gradually reacted with an excess of iodide to form I3 with an absorption maximum of 365 nm. The concentration of lipoperoxides was given in nmol/mL.
The concentration of advanced protein oxidation products (AOPP) was determined at 340 nm, according to the method of Witko-Sarsat et al., based on a calibration curve of chloramine T with potassium iodide. The AOPP concentration was given in mol/L.
Plasma nitrotyrosine (NT) was determined by the Nitrotyrosine Elisa Kit (Hycult biotech, No. HK 501-02, 5405 PB, Uden, Netherlands). The nitrotyrosine concentration was given in nmol/L.
The Trolox equivalent antioxidant capacity of serum (TEAC) assay measured the antioxidant ability to scavenge the stable radical cation ABTS+ (2,20-azinobis(3-ethylbenzothiazoline-6-sulfonic acid). A blue-green chromophore with maximum absorption at 734 nm was

decolorised in the presence of both lipophilic and hydrophilic hydrogen-donating antioxidants. The antioxidant activity of the sample was compared with the antioxidant activity of the synthetic vitamin E, Trolox (Re et al.). The concentration of Trolox equivalents was expressed in mmol/L.

Glutathione peroxidase (GPx) activity was determined in erythrocyte hemolysates using Glutathione Peroxidase Activity Kit (Enzo Life Sciences, No. ADI-900-158, CH-4415 Lausen, Switzerland, Biotech distributor), according to the manufacturer's protocols. The activity was given in cat/mg of Hb.

Creatinine in the urine was determined at the Department of Clinical Biochemistry of National Institute of Children's Diseases using a Hitachi 911 analyzer by a standard procedure using Roche Diagnostics kits (Roche Diagnostics International, Bratislava, Slovakia). The concentration was given in mmol/L (22).

El–Saadani, M.; Esterbauer, H.; El-Sayed, M.; Goher, M.; Nassar, A.Y.; Jürgens, G. A spectrophotometric assay for lipid peroxides in serum lipoproteins using a commercially available reagent. J. Lipid Res. **1989**, 30, 627–630.

Witko-Sarsat, V.; Friedlander, M.; Capeillere-Blandin, C.; Nguyen-Khoa, T.; Nguyen, A.T.; Zingraff, J.; Jungers, P.;
Descamps-Latscha, B. AOPP as a novel marker of oxidative stress in uremia. Kidney Int. **1996**, 49, 1304–1313.

Re, E.; Pellegrini, L.; Prottegente, A.; Pannala, A.; Yang, M.; Rice-Evans, C. Antioxidant activity applying an improved ABTS radical cation decolorization assay. Free Radic. Biol. Med. **1999**, 9–10, 1231–1237.

5. Determination of cortisol

Cortisol concentrations in saliva were determined using a commercially available enzyme-linked immunosorbent assay (IBL International, Hamburg, Germany). The intra- and inter-assay coefficients of variation were 2.9% and 5.0%, respectively (15).
