# Supplementary material for: The kynurenine and serotonin pathway, neopterin and biopterin in depressed children and adolescents: an impact of omega-3 fatty acids, and association with markers related to depressive disorder. A randomized, blinded, prospective study
Source: Front Psychiatry. 2024 Feb 13;15:1347178. doi: 10.3389/fpsyt.2024.1347178 (PMC10896889; doi:10.3389/fpsyt.2024.1347178)
Supplement: Supplementary file 3 [file DataSheet_3.pdf]

1. **Study design and intervention**

Patients were randomized to receive either an omega-3 FA-rich fish oil emulsion (Om3 group) or an active comparator of omega-6 FA-rich sunflower oil emulsion (Om6 group) for 12 weeks. Children were included in the study according to ICD 10 with the diagnosis of depressive disorder (DD, Dg F32.0, F32.1, F32.2, n = 31; 51.7%) or mixed anxiety and depressive disorder (MADD, Dg F41.2, n =29; 48.3%).

Compliance to the product was assessed by monitoring the volume of intervention oil returned and was above 95%.

2. **Randomisation**

Trial participants were allocated in a 1:1 ratio to the two arms (Om3 and Om6) according to a computer-generated random sequence using block randomization with a block size of four. The randomization was performed by an independent statistician. Patients were enrolled and assigned sequentially to supplement interventions by the physician. The allocation sequence was not available to any member of the research team until the databases had been completed and locked.

3. **Enrolment and baseline characteristics**

Patient characteristics such as age (15.7±1.6 years, 10 – 18 years), gender, weight, height, and body mass index (BMI) (weight (kg) / (height (m)^2) are in Table S1.

From the 60 patients included in our study, two dropped out at an early stage (after one-two day after the enrolment); one patient from the Omega-3 group due to the product palatability and one from the Om6 group for non-compliance (reluctance to miss school every two weeks to visit the clinic, difficulties with transportation of outside city patient).

Of the 58 patients who completed the intervention, 29 patients (21 F and 8 M) were included in the data analysis in the Om3 group and 29 patients in the Om6 group (25 F and 4 M). Improvement in depressive symptoms was rated as CDI. In parallel, all randomized subjects were analyzed according to randomization.

Patients consumed a standard diet and were advised to consult or report any change in the diet during the intervention.

Both emulsions were well tolerated, and no serious adverse side effects were recorded. Only one patient from the Om3 group stated more frequent defecation (2-3× daily).

The number of patients who left the study, and the proportion of diagnoses are shown in the Flow Diagram (Supplement 1).

4. Clinical investigation

Patient characteristics (age, gender, menstruation in females) and relevant clinical variables (treatment history – duration of the disease/firstly diagnosed. treatment/no treatment. current medication with antidepressants) were recorded for each patient.

Clinical examinations of all participants were implemented as follows: at the beginning of the trial (week 0) and every 2 weeks for 3 months (weeks 2, 4, 6, 8, 10, 12). The process of data collection is graphically depicted in a Consort flow diagram (Supplement 1).

5. Sample size estimation (Power analysis)

A number of patients enrolled in the project was calculated using the statistical program StatsDirect as a randomized intervention study by t-test with the choice of parameters: Estimated effect (difference between means - effect size) - score 5 points. Estimation of the population deviation (standard deviation) – 6, balanced design - 1:1, the test power - 80%, Alpha level - 5%. The number of patients in each group (control and intervention) was set at 19. Assuming that some patients might leave the project, we increased the number to 30 patients.
